# Cervial artery dissection: value of 3D high resolution vessel wall magnetic resonance imaging for diagnosis and follow-up

**DOI:** 10.1186/1532-429X-17-S1-P412

**Published:** 2015-02-03

**Authors:** Qi Yang, Xiuhai Guo, Zhaoyang Fan, Min Wan, Debiao Li

**Affiliations:** 1 Biomedical Imaging Research Institute, Cedars Sinai Medical Center, Los Angeles, CA, USA; 2 Xuanwu Hospital, Beijing, China

## Background

Cervical artery dissection (CD) is an important cause of stroke in young and middle-aged patients. Sampling perfection with application-optimised contrast using different flip angle evolutions (SPACE) sequence can provide fast 3D isotropic T1 FS images, with very good dark blood contrast. This study aims to demonstrate the value of a 3D fat-saturated SPACE for the diagnosis and follow up of CD.

## Methods

Twenty-one patients were prospectively evaluated on a 3.0-T MR system for a clinical suspicion of acute or sub-acute cervical artery dissection with 3D T1 SPACE sequence. All scans were performed with the routine head and neck coil. The HR-MRI protocol contained at least three sequences: 3D-TOF, 2D-T1WI and 3D-SPACE. For each patient, coronal, oblique and/or curvilinear multiplanar reconstructions were generated from 3D T1 SPACE datasets. These patients underwent HR-MRI every three months until the double-lumen sign disappeared or until the patients had no appreciable changes for two consecutive times of scanning or turned to operation due to deterioration. The final diagnosis was established in consensus, after reviewing all the imaging and clinical data.

## Results

**Figure 1:**
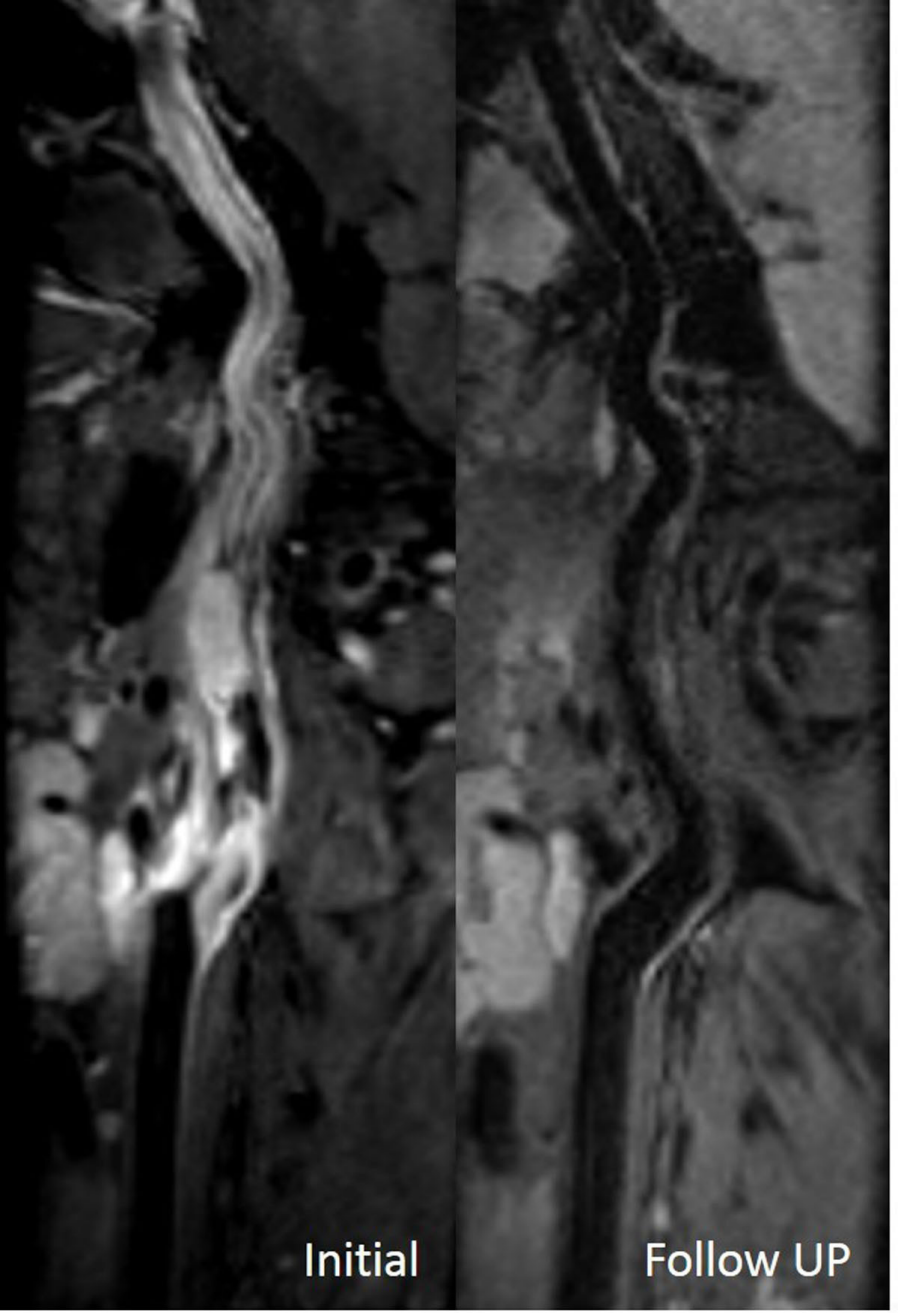
**A 29-year old male presented with slurred speech and weakness of right limbs.** Curved MPR images of initial T1 SPACE shows the subacute mural hematoma in the left carotid artery. Three-month follow-up shows resolution of hemorrhage on T1 SPACE.

Final diagnosis of acute or subacute dissection was established in 11 patients. Overall sensitivity and specificity were 0.955 and 0.965 for T1 SPACE in the detection of dissection. T1 SPACE has reduced imaging time compared with 2D TSE(P<0.05). In eight CD patients, follow-up MRI has shown that intramural hematomas completely resolved (Figure 1).

## Conclusions

3D SPACE sequence offers similar information with its 2D counterpart, in a shorter acquisition time and larger coverage area. Multiplanar reconstructions were very useful in tortuous regions, such as the atlas loop of the vertebral artery or the carotid petrous entry which was missed by 2D-TSE. MRI would be a reliable mean for the diagnosis and follow-up of CD.

## Funding

This work has been supported by NSFC 81322022.

